# A Polymorphism in the Catalase Gene Promoter Confers Protection against Severe RSV Bronchiolitis

**DOI:** 10.3390/v12010057

**Published:** 2020-01-03

**Authors:** Jeffrey M. Chambliss, Maria Ansar, John P. Kelley, Heidi Spratt, Roberto P. Garofalo, Antonella Casola

**Affiliations:** 1 Department of Internal Medicine, UT Southwestern Medical Center, Dallas, TX 75390, USA; jeffrey.chambliss@utsouthwestern.edu; 2 Department of Microbiology and Immunology, The University of Texas Medical Branch at Galveston, Galveston, TX 77555, USA; maansar@utmb.edu (M.A.); rpgarofa@utmb.edu (R.P.G.); 3 Southwest Asthma and Allergy Associates, Houston, TX 77074, USA; jpkelley@gmail.com; 4 Department of Preventative Medicine and Community Health, The University of Texas Medical Branch at Galveston, Galveston, TX 77555 USA; hespratt@utmb.edu; 5 Department of Pediatrics, The University of Texas Medical Branch at Galveston, Galveston, TX 77555, USA

**Keywords:** pneumovirus, lung disease, bronchiolitis, catalase, polymorphism

## Abstract

Respiratory syncytial virus (RSV) infection is associated with oxidative lung injury, decreased levels of antioxidant enzymes (AOEs), and the degradation of the transcription factor NF-E2-related factor 2 (NRF2), a master regulator of AOE expression. Single nucleotide polymorphisms (SNPs) in AOE and NRF2 genes have been associated with various lung disorders. To test whether specific NRF2 and/or AOE gene SNPs in children with RSV lower respiratory tract infection were associated with disease severity, one hundred and forty one children <24 month of age with bronchiolitis were assessed for seven AOE and two NRF2 SNPs, and data were correlated with disease severity, which was determined by need of oxygen supplementation and intensive care support. One SNP in the promoter region of the catalase gene, rs1001179, which is associated with higher enzyme expression, was significantly underrepresented (*p* = 0.01, OR 0.38) among patients with moderate to severe RSV bronchiolitis, suggesting a protective effect against disease severity. Our results suggest that increasing catalase expression/activity could exert a protective role in the context of RSV infection and represent a potential novel therapeutic target to ameliorate viral-induced lung disease.

## 1. Introduction

Respiratory syncytial virus (RSV) bronchiolitis remains one of the most common respiratory infections in infants and young children. It resulted in an estimated 33.1 million cases of lower respiratory tract infection (LRTIs) globally in 2015, accounting for 3.2 million hospital admissions children [1]. There is significant morbidity and mortality associated with severe RSV bronchiolitis, particularly in developing countries [2]. While the use of palivizumab, a monoclonal antibody, is fairly effective for RSV infection prophylaxis in high risk patients, current management is otherwise supportive [3]. Our understanding of the underlying molecular mechanisms that are responsible for RSV lower respiratory tract disease is still incomplete and remains a critical need to improve patient care and outcomes. Previous work in our laboratory has shown that RSV infection is associated with increased reactive oxygen species (ROS) production, leading to transcription factor activation and chemokine gene expression [4,5]. The transcription factor NF-E2-related factor 2 (NRF2), which binds the antioxidant response element (ARE) present in the promoter region of antioxidant enzymes (AOEs), plays a key role in the expression of AOEs [6]. NRF2 cellular levels decrease in the course of RSV infection in vitro [7] and in vivo [8] due to increased degradation [9,10], leading to the inhibition of AOE expression and cellular oxidative damage [7,8], a process that has been implicated in the pathogenesis of acute and chronic airway diseases [11].

Single nucleotide polymorphisms (SNPs) are the most common source of genetic variation among humans, and, when present within or in proximity of a gene, may impact gene function. SNPs have been associated with airway diseases including asthma and COPD [12,13], but knowledge of their contribution to virus-associated lung disorders is limited. In this study, we focused on SNPs of AOEs and NRF2 that had previously been evaluated in the context of acute lung injury, asthma, and COPD exacerbations [14,15,16]. Of the evaluated SNPs that is found in the promoter region of catalase gene, thus resulting in higher enzyme expression, was found to be remarkably underrepresented among patients with moderate to severe RSV bronchiolitis compared to patients with milder forms of the disease. Some of the results of this study have previously been presented as an abstract [17].

## 2. Materials and Methods

### 2.1. Study Population

The children included in the present analysis were enrolled in an ongoing study of the pathogenesis of RSV bronchiolitis from October 2013 to March 2017, thus covering four distinct RSV seasons. Protocol #03-117 was approved on 6 April 2016 by the Institutional Review Board of UTMB, and informed written consent was obtained from the parents or custodians of all participants. The study population included children ≤2 years of age who were either admitted to the UTMB pediatric inpatient unit or presented at outpatient pediatric clinics with symptoms of LRTI. Samples of nasopharyngeal secretions (NPS) were collected from children, as previously described [18,19,20]. NPS samples were used for RSV and other virus identification, as well as for DNA extraction, by using the Luminex NxTAG™ Respiratory Pathogen Panel (RPP, Luminex Molecular Diagnostics, Austin, TX, USA). RSV-positive samples were further genotyped for SNPs. Patients were retrospectively categorized into three disease severity groups based on their requirement for oxygen supplementation; mild: none; moderate: ≥12 h of supplemental oxygen; and severe: requiring admission to the pediatric intensive care unit (PICU). The mild group included both hospitalized and non-hospitalized patients. Additional patient information, including demographic data and other clinical characteristics, was retrospectively reviewed and is presented in Table 1. The median PICU stay was three days. Fisher’s exact test was used to assess whether there were differences between severity groups in the number of premature babies (<38 weeks at birth), as well as with smoking exposure (not determined, absent, present).

### 2.2. Genotyping for SNPs

Samples were analyzed for preselected SNPs in NRF2 and NRF2-dependent genes at the Genetic Resources Core Facility, Johns Hopkins University, Baltimore, MD, USA. The specific nine SNPs analyzed are shown in Table 2. The genotyping of SNPs rs1001179, rs1806649, rs1695, rs4880, rs1138272, rs7943316, rs769217 was carried out with the use of pre-designed TaqMan^®^ Assays C__11468118_10, C__11634983_10, C___3237198_20, C___8709053_10, C___1049615_20, and C___1883210_10, C___3102907_10, respectively, (Applied Biosystems, Foster City, CA, USA) following the manufacturer’s supplied protocols. SNP rs1050450 was genotyped with the use of a custom TaqMan assay by using the forward primer CATCGAAGCCCTGCTGTCT, the reverse primer CACTGCAACTGCCAAGCA, VIC probe CAGCTGGGCCCTTG and FAM probe CAGCTGAGCCCTTG following the manufacturer’s protocols. PCR and the endpoint detection of fluorescence was carried out in an ABI Prism7900HT Sequence Detection System (Applied Biosystems, Foster City, CA, USA) with default settings. Fluorescence data were analyzed with the ABI Prism 7900 allelic discrimination software. SNP rs6721961 analysis was performed with a custom pyrosequencing assay. PCR was carried out with a PyroMark PCR Kit (Qiagen, Valencia, CA, USA) following the manufacturer’s protocols.

### 2.3. Catalase Activity

A catalase activity assay kit from Cell Biolabs (San Diego, CA, USA) was utilized to measure catalase activity in human NPS samples after protein/volume normalization.

### 2.4. Statistical Analysis

To analyze the effects of SNPs on disease severity, each SNP was individually evaluated in regard to its presence in severity groups, comparing mild patients to hypoxemic patients (moderate-plus-severe patients). The less frequent genotypes were combined, and the Chi-square test was used to analyze the data. Calculations were made for the combined cohort as well as individual races. In addition, odds ratios were calculated for the mild versus moderate-plus-severe clinical phenotypes, comparing the more common to the less common genotype. The method of Benjamini–Hochberg was used to correct for multiple hypothesis testing, and q-values are reported in Table 2. A comparison of expected frequency by race versus the actual frequency in our overall study population was performed by using an exact goodness of fit test via a binomial test. A similar test was performed to compare rs1001179 allele frequency in the mild or moderate-plus-severe patient group to the expected population frequency, according to the individual race. This test is appropriate for a variable with multiple categories and is useful to determine whether the number of observations in each category matches a specified value. Due to a lack of power, a multivariate analysis was not performed to examine the effect of the SNP on catalase activity and RSV severity by gestational age, age at the time of infection, and second hand smoke.

The expected prevalence of catalase SNP rs1001179 in different races was taken from the 1000 Genome Project Phase 3 (http://www.internationalgenome.org/1000-genomes-browsers). All human subjects data were analyzed in R (version 3.5.0) with a significance level of 0.05.

## 3. Results

Catalase SNP rs1001179 is protective against RSV severe disease. The baseline demographics of the study population are shown in Table 1. A total of 136 patients with RSV-positive LRTI were genotyped for seven AOE and two NRF2 SNPs, which were selected based on their potential association with lung diseases (Table 2). The study population included 64 children with mild and 72 with combined moderate/severe LRTI, of which 27% were African American, 49% were Caucasian, and 24% were Hispanic. There were slightly more males than females in each of the severity groups. The median age decreased with severity of illness from nine months in the mild group to three months in the severe group. Fisher’s exact tests of the three disease severity groups indicated that there was a difference between groups in number of patients born before 38 weeks of gestation, while a similar test for smoking exposure indicated that there was no difference amongst them. In regard to respiratory viruses that are associated with LRTI, 30% of the patients were RSV-A-positive only, 27% were RSV-B-positive only, and the remaining 43% were found to be positive for RSV and one or more additional viruses.

A comparison between the group of patients with mild disease versus the combined groups of patients with a diagnosis of moderate/severe disease showed that, among the nine SNPs investigated, only catalase rs1001179 reached statistical significance because it was significantly underrepresented among the hypoxemic patients [(*p* = 0.012 with an OR of 0.38 (Table 2)]. The same polymorphism also showed a trend in being underrepresented among the hypoxemic patients when correcting for multiple hypothesis testing, although it did not reach statistical significance.

The rs1001179 was mapped to the catalase gene promoter, and it was defined by a C-to-T substitution at position −262. This polymorphism enhances catalase gene transcription, as carriers of the −262T allele display an increased basal catalase expression in various cell types [21]. The prevalence of rs1001179 varies among different races, but CC is the more common genotype in all races, followed by CT and then TT. By comparing the rs1001179 genotype racial distribution in our study population with the expected general prevalence, we found that there was a statistically significant difference for the Caucasian and Hispanic patients, but there was not a statistically significant difference for the African American patients (Table 3).

When the frequency by race for rs1001179 alleles was compared between the expected one in the general population and our disease groups, no statistically significant difference for all races was observed in the patient group with the mild form of the disease. On the other hand, allele distribution was significantly different in the combined moderate/severe disease groups of Caucasians and Hispanics patients, with a progressive increase of the CC allele and a parallel decrease of the CT/TT alleles (Table 4). Overall, the CT/TT genotypes were absent in all 15 patients that had the most severe forms of RSV LRTI (i.e., those requiring intensive care unit admission).

**Figure 1:**
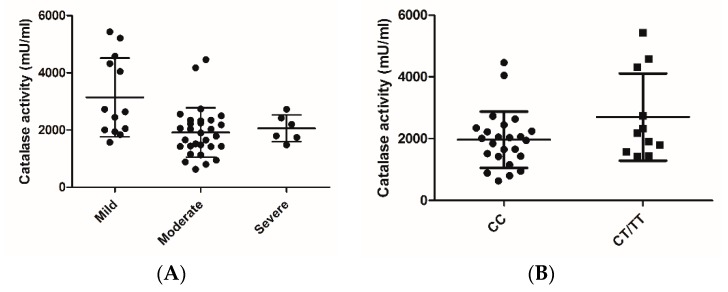
(**A**) Catalase activity in nasopharyngeal secretions (NPS) of infants with respiratory syncytial virus (RSV) lower respiratory tract infections (LRTI) of different severities (mild, moderate, severe). NPS obtained from RSV-positive LRTI patients were tested for catalase activity. Assays were performed on samples in a blinded manner by using numerical identifiers, and activity was determined after volume/protein normalization. Statistics were determined by using a one-way ANOVA, with *p* < 0.05 for moderate-plus-severe vs mild. (**B**) Catalase activity in the NPS of infants with RSV LRTI according to the presence of the CC or CT/TT allele. Statistics were determined by using one tailed t-test with Welch’s correction.

A measurement of catalase activity in NPS samples showed that the patients in the group with the moderate or severe LRT RSV disease had significant lower catalase activity compared to those in the mild group (Figure 1A). A comparison of catalase activity in NPS of infants with RSV LRTI according to the presence of the rs1001179 CC or CT/TT alleles showed a trend towards higher activity in the CT/TT group; however, this did not reach statistical significance (*p <* 0.07).

## 4. Discussion

Catalase is an antioxidant enzyme that is present in most aerobic cells and that catalyzes the dismutation of hydrogen peroxide to water and oxygen [22]. In the lung, catalase activity increases in parallel with the its embryologic development, and, at full maturity, it is specially localized in alveolar type II pneumocytes and alveolar macrophages, with both cell types being a primary target of respiratory viral entry and replication [23]. While certain stimuli such as hyperoxia and cytokines induce catalase expression/activity, other stimuli like prolonged cigarette smoke exposure can lead to decreased catalase mRNA and protein levels [24,25]. In addition, genetic factors such as polymorphisms in the catalase gene promoter have been associated with the increased basal expression of catalase gene, which may also contribute to an enhancement in its overall anti-oxidative capacity [21]. However, to the best of our knowledge, catalase gene polymorphisms in either the promoter or coding region have not been evaluated in the context of respiratory infections.

The results of our study suggest that lung catalase may play an important and previously unrecognized protective role in RSV-associated LRTIs. Infants and young children carrying the genotypes of the catalase SNP rs1001179 with the CT/TT allele were found to have significantly less severe RSV bronchiolitis in our study population, suggesting a protective effect on disease severity. As such, the genotypes containing at least one T allele were represented near the expected population frequency among Caucasian and Hispanic patients that presented with mild forms of RSV LRTI, while T alleles were significantly less common among those with moderate and severe forms of the disease. Though our results suggest a newly described association of catalase rs1001179 with decreased RSV bronchiolitis severity, co-morbidities and other predisposing genetic factors are likely to play an important role in patients with the CT/TT genotype who present with different illness severities. At the functional level, rs1001179 (−262C/T) may influence catalase gene transcription by affecting transcription factor binding, and carriers of the less common −262T allele have higher amounts of catalase in different types of cells, including erythrocytes and in whole blood [26,27]. In a small pilot study of RSV-infected infants, we previously found that the protein levels of AOEs, including catalase, were reduced in NPS samples that were obtained from hypoxemic patients—in particular those requiring ventilatory support—compared to patients with mild disease [8]. Our current result of decreased catalase enzymatic activity in patients with a more severe disease (Figure 1A) is in agreement with those earlier findings. Our analysis of the catalase activity in NPS of infants with RSV LRTI according to the presence of the rs1001179 CC or CT/TT allele showed a trend towards higher activity in the CT/TT group (Figure 1B); however, this trend did not reach statistical significance, possibly due to the limited number of patients who carried the minor allele.

In the context of airway diseases and in different ethnic groups, the presence of the T allele has been previously associated with both a decreased risk of asthma in a non-smoker, Hong Kong Chinese population [28] and with increased risk of new-onset of asthma among Hispanic and white children [29]. Given the still not fully understood link between RSV LRTI in infancy and the development of asthma later in life, it may therefore be of interest to consider future longitudinal studies to address the possible contribution of the catalase SNP rs1001179 as a risk factor for the development of asthma in this population.

## 5. Conclusions

In summary, our results suggest that the antioxidant catalase exerts a protective role in the context of RSV infection. A larger sample study will be needed to confirm our initial findings, including the correlation of catalase activity with the specific polymorphic allele. Increasing airway catalase levels at the time of RSV infection could represent a potential novel therapeutic approach to ameliorate viral-induced lung disease.

## Figures and Tables

**Table 1:** Baseline demographics by disease severity.

	All		Mild		Moderate and Severe		Moderate Only		Severe Only	
	*n* = 136		*n* = 64		*n* = 72		*n* = 57		*n* = 15	
**Median Age, Months**	6		9		4		5		3	
**Gender**										
Males: *n* (%)	86	63.2%	40	62.5%	46	63.9%	37	64.9%	9	60.0%
Females: *n* (%)	50	36.7%	24	37.5%	26	36.1%	20	35.1%	6	40.0%
**Race**										
Caucasian: *n* (%)	67	49.3%	33	51.6%	34	47.2%	27	47.4%	7	46.7%
Hispanic: *n* (%)	37	27.2%	14	21.9%	23	31.9%	20	37.1%	3	20.0%
African American: *n* (%)	32	23.5%	17	26.7%	15	20.8%	10	17.5%	5	33.3%
**Median Length of Hospital Stay (Days)**	2		1		6		5		8	
**Percent Hospitalized**			64.1%		100%					
**Preterm (<38 Weeks Gestation): *n* (%)**	21	15.5%	2	3%	19	26.4%	15	26.3%	4	26.7%
**Smoke Exposure: *n* (%)**										
Yes: *n* (%)	28	20.6%	11	17.2%	17	23.6%	11	19.3%	6	40.0%
No: *n* (%)	89	65.5%	40	62.5%	49	68.1%	40	70.2%	9	60.0%
Not documented: *n* (%)	19	14.0%	13	20.3%	6	8.3%	6	10.5%	0	0.0%
**Personal Atopic History**										
Yes: *n* (%)	34	25.0%	15	23.4%	19	26.4%	17	29.8%	2	13.3%
No: *n* (%)	102	75.0%	49	76.6%	53	73.6%	40	70.2%	13	86.7%
**Atopic Family History**										
Yes: *n* (%)	28	20.6%	13	20.3%	15	20.8%	11	19.3%	4	26.7%
No: *n* (%)	73	53.7%	28	43.8%	45	62.5%	36	63.2%	9	60.0%
Not documented: *n* (%)	35	25.7%	23	35.9%	12	16.7%	10	17.6%	2	13.3%

Fisher’s exact test was used to assess differences between severity groups in the number of premature babies and smoking exposure. *p* < 0.05 only for number of premature babies.

**Table 2:** Single nucleotide polymorphisms (SNPs) analyzed for association with disease severity.

SNPs Analyzed		*p*-Value	Odd Ratio	BH Q-Value
Catalase	rs1001179	0.0122	0.38 (0.14, 0.93)	0.0854
Glutathione peroxidase 1	rs1050450	1	0.99 (0.5, 1.96)	1
Glutathione S-transferase P	rs1695	0.617	1.28 (0.63, 2.61)	1
	rs1138272	1	1.18 (0.3, 5)	1
NRF2 promoter	rs6721961	0.385	0.65 (0.29, 1.43)	1
NRF2 intron	rs1806649	0.561	1.37 (0.62, 3.14)	1
Superoxide dismutase 2	rs4880	1	0.97 (0.49, 1.92)	1

Comparison was made between mild versus moderate plus severe disease, *p*-value was determined by Chi-square test. Definition of abbreviations: NF-E2-related factor 2, NRF2.

**Table 3:** Comparison of catalase SNP rs1001179 prevalence by race in the general population versus our study population.

	African American Population	Study Sample	*p*-Value	Caucasian Population	Study Sample	*p*-Value	Hispanic Population	Study Sample	*p*-Value
CC	90.2%	90.6%	NS	54.5%	74.6%	<0.001	71.9%	89.2%	0.017
CT/TT	9.8%	9.4%		45.5%	25.4%		28.1%	10.8%	

The expected prevalence of catalase SNP rs1001179 in different races was taken from the 1000 Genome Project, and the *p*-value was determined by an exact goodness of fit test via a binomial test. NS: non-significant.

**Table 4:** Distribution of rs1001179 by race and disease severity.

**African American, *n* = 32**	**CC**		***p*-Value**	**CT/TT**	
Mild	16	94%	NS	1	6%
Moderate and Severe	13	87%	NS	2	13%
Severe only	5	100%		0	0%
**Caucasian, *n* = 67**					
Mild	20	61%	NS	13	39%
Moderate and Severe	30	88%	4.0 × 10^−5^	4	12%
Severe only	7	100%		0	0%
**Hispanic, *n* = 37**					
Mild	12	86%	NS	2	14%
Moderate and Severe	21	91%	0.03	2	9%
Severe only	3	100%		0	0%

A comparison was made between the expected allele frequency for a specific race and the frequency of mild or combined moderate-plus-severe disease group of the same race. The *p*-value was determined by an exact goodness of fit test via a binomial test. NS: non-significant.
